# Totally transaxillary endoscopic surgical release for congenital muscular torticollis

**DOI:** 10.3389/fsurg.2025.1507251

**Published:** 2025-11-17

**Authors:** Junjie Sun, Hao Chen, Zhihai Zhong, Qigen Xie, Wenzong Gao, Hong Jiang, Yunjie Yang, Pengfei Gao

**Affiliations:** 1 Department of Pediatric Surgery, Shenzhen Children’s Hospital, Shenzhen, China; 2 Department of Pediatric Surgery, Quanzhou First Hospital Affiliated to Fujian Medical University, Quanzhou, China; 3 Department of Pediatric Surgery, The First Affiliated Hospital, Sun Yat-sen University, Guangzhou, China; 4 Department of Pediatric Surgery, The Sixth Affiliated Hospital, South China University of Technology, Foshan, China

**Keywords:** congenital muscular torticollis, endoscopic surgery, open surgery, minimally invasive approach, cosmetic result

## Abstract

**Introduction:**

This study aims to present our experience with totally transaxillary endoscopic surgical release (TTESR) for the treatment of patients diagnosed with congenital muscular torticollis (CMT), as well as to compare the efficacy of this minimally invasive approach with that of conventional open surgical release (OSR).

**Materials and methods:**

A retrospective analysis was conducted on patients diagnosed with CMT who underwent either TTESR or OSR between January 2014 and December 2020. Herein, we provide a detailed description of the TTESR procedure. A total of 24 children were enrolled, with 6 patients undergoing TTESR and the remaining 18 undergoing OSR. The latter group was matched based on age and lesion location. Clinical data, including length of hospital stay, operative duration, intraoperative blood loss, and range of cervical rotation, were meticulously recorded. Comparative analysis was performed between the TTESR and OSR groups.

**Results:**

In our series, all 24 patients exhibited a marked improvement in cervical range of motion. No statistically significant differences were observed between the TTESR and OSR groups with respect to gender distribution, length of hospital stay, operative duration, or intraoperative blood loss. However, a significant difference was noted in the combined scores of scar evaluation and subjective assessments. No severe postoperative complications were reported. Additionally, with the accumulation of surgical experience, the average operative time for TTESR decreased to 40–50 min.

**Conclusion:**

This study demonstrates that TTESR serves an effective alternative to conventional OSR for correcting CMT, with the additional advantage of eliminating the aesthetically undesirable neck scar.

## Introduction

Congenital muscular torticollis (CMT) is a common congenital abnormality in pediatric populations, characterized by contracture of the sternocleidomastoid (SCM) muscle. This condition leads to restricted neck movement, facial asymmetry, heterotropia, cranial plagiocephaly and postural compensation (1, 2). The incidence rate of CMT varies between 0.3% to 3.92%, with a noted increasing trend (1, 3). Although multiple etiological theories have been proposed, the precise cause of CMT remains elusive (4). Physical therapy, as a conservative treatment approach, has demonstrated efficacy for infants within their first year of life (5, 6). However, a subset of patients who undergo physical therapy beyond six months or are older than one year may exhibit refractory and rigid symptoms (7). In these cases, surgical release of the contracted SCM muscle is considered a necessary intervention.

Conventionally, CMT has been managed through open surgical release (OSR), which leaves a visible transverse scar on the neck. This scar often leads to dissatisfaction among parents. With the advancement of minimally invasive surgical techniques, several procedures have been reported in the literature (8). Nevertheless, the existing research lacks comprehensive comparative studies to evaluate these approaches.

This study provides a detailed and systematic guide to the totally transaxillary endoscopic surgical release (TTESR) for the treatment of CMT, emphasizing critical tips and techniques at each stage of the procedure. Additionally, this study evaluates the efficacy of TTESR in comparison to OSR and demonstrates our learning curves associated with TTESR.

## Materials and methods

A retrospective cohort study was conducted, involving clinical follow-up of patients diagnosed with CMT who underwent surgical treatment between January 2014 and December 2020. The diagnosis of CMT was confirmed through a comprehensive physical examination and ultrasonographic evaluation to ensure accurate differentiation from other conditions. Any abnormalities associated with the cervical spine or torticollis caused by ocular factors were systematically excluded. This study was approved by the Institutional Review Board of the First Affiliated Hospital of Sun Yat-sen University (IIT-2023-269). Due to the retrospective nature of the study, the need for informed patient consent was formally waived.

TTESR was performed on 6 patients by the same surgical team led by Dr. Sun. This study included 18 children with OSR, who were selected based on age and lesion site matching criteria and subsequently treated by other surgical teams. Abnormal head positioning was observed in all patients, leading to their evaluation as candidates for surgical intervention. A neck splint was employed in the postoperative period. The clinical data collected and analyzed included gender, age, lesion location, length of hospital stay, operative duration, intraoperative blood loss, scar scoring and subjective assessments, as well as overall result scoring. In our cohort, subjective assessments commonly focused on factors such as whether wound infection or visibility.

The clinical assessments were evaluated using the modified Cheng and Tang Scoring System (9, 10), which incorporated both subjective and objective criteria. These criteria included rotation deficit, lateral bending deficit, craniofacial asymmetry, residual contracture, head tilt, and subjective evaluation. The modified Cheng and Tang scoring system is standardized on a scale ranging from 0 to 21 points. Patients are classified into four categories based on their scores: excellent outcomes (17–21 points), good outcomes (12–16 points), fair outcomes (7–11 points), and poor outcomes (<7 points) (9, 10).

## TTESR technique

**Figure 1:**
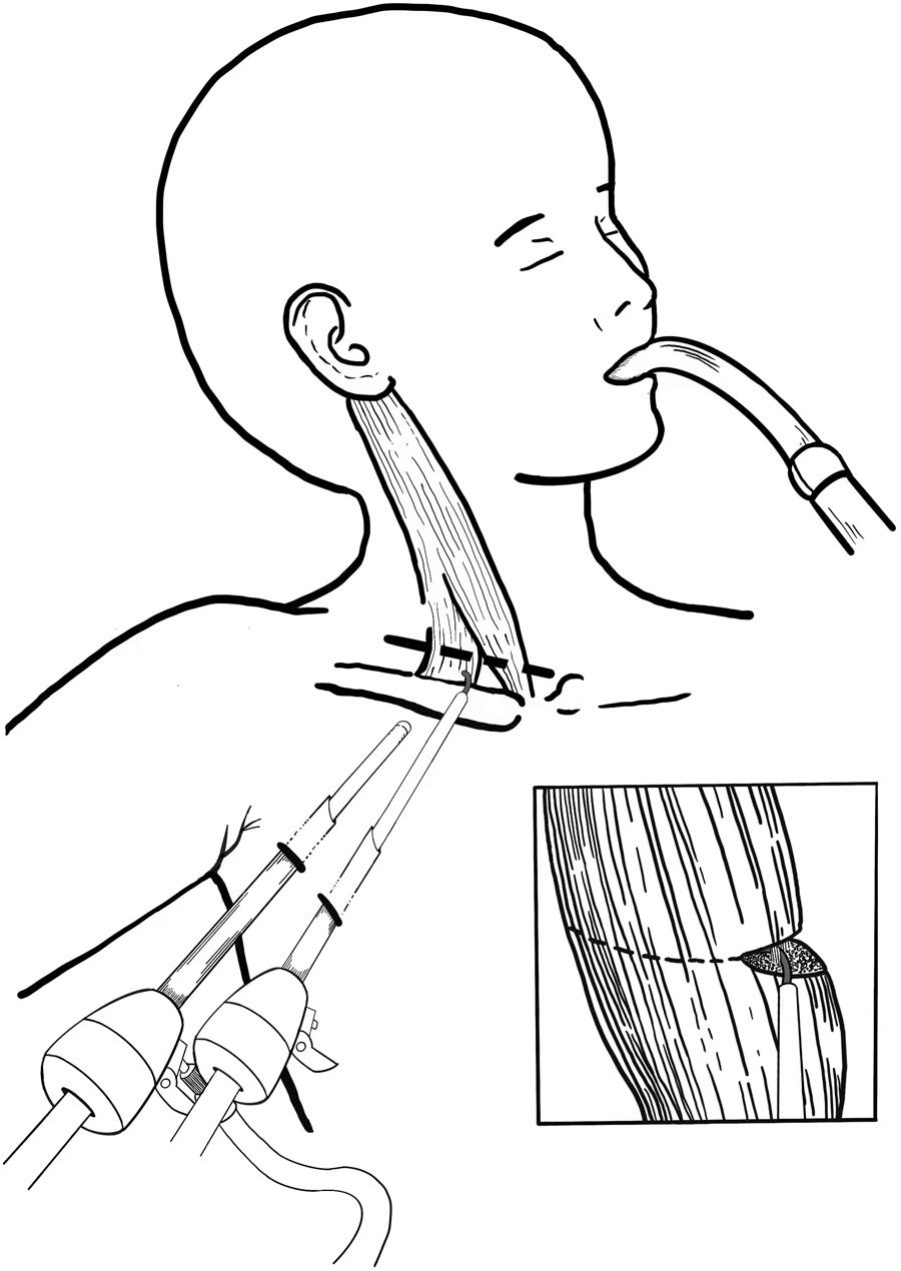
Schematic illustration of TTESR.

**Figure 2:**
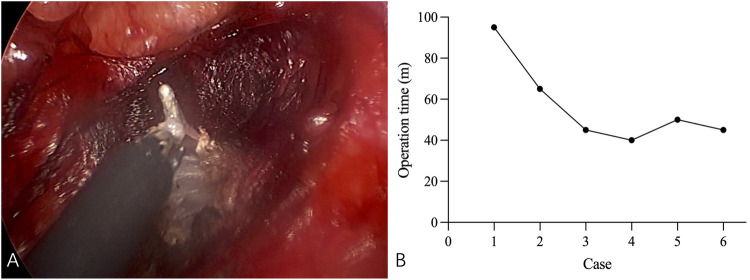
**(A)** The SCM muscle was released using electrocautery, fascicle by fascicle. **(B)** Operative time of 6 patients who underwent TTESR.

General anesthesia with tracheal intubation was administered for the procedure. Patients were positioned with their head and neck rotated contralaterally to the lesion site, and their ipsilateral arm abducted at approximately 90 degrees. Preoperatively, the clavicular and sternal heads of the SCM muscle, as well as the clavicle, were clearly marked. Two incisions were made along the anterior axillary line, a tunnel and subcutaneous working space were created via blunt dissection beneath the platysma muscle using forceps through one incision. Trocars were subsequently inserted. The endoscope was introduced through the 5 mm incision, while electrocautery was applied via the 3 mm incision (Figure 1). During the procedure, the sternal and clavicular heads of the SCM muscle were consistently identified through frequent transdermal palpation to facilitate precise navigation and dissection. The subcutaneous space was maintained with CO_2_ insufflation at a pressure of 6 mmHg and a flow rate of 4–5 L/min. The sternal and clavicular heads of the SCM muscle were carefully released fascicle by fascicle using electrocautery until no residual fibrotic bands or tightness were observed (Figure 2A). The operator meticulously coagulated all potential bleeding points to prevent hematoma formation. Particular emphasis was placed on clearly visualizing and carefully exposing the adjacent carotid sheath and neural tracts to ensure their protection and avoid potential damage. Following the complete dissection of the muscle, the trocars were systematically removed, and both incisions were closed using absorbable sutures. A compression dressing was subsequently applied to the subcutaneous tunnel. All patients were instructed to perform basic postural exercises and active stretching routines aimed at preventing re-adhesion and contracture. Additionally, the postoperative use of a neck splint was incorporated into the rehabilitation protocol. Furthermore, the 3 mm trocar was maintained in a partially open state during the procedure, facilitating efficient smoke evacuation while preserving the integrity of the surgical field.

## Statistical analysis

Data were stored and analyzed using SPSS software (version 26.0 for Mac). Continuous variables were presented as mean ± standard deviation or median with range, while categorical variables were reported as counts and percentages. Comparisons of continuous data were performed using either the independent samples *t*-test or the Mann–Whitney U test, depending on the distribution of the data. Categorical variables were analyzed using either the Chi-square test or Fisher's exact test, as appropriate. A significance level of *P* < 0.05 was set for all analyses.

## Results

The TTESR group demonstrated excellent tolerance and experienced a lack of complications during the procedure. The characteristics and comparison between the TTESR group and the OSR group are summarized in Table 1. No significant differences were observed between the two groups regarding age, gender, location site, length of hospital stay, operative time, intraoperative blood loss, or overall scoring. The only statistically significant difference between the two groups was observed in the scores based on the sum of scar and subjective assessments.

**Table 1:** The characteristics and comparison between the TTESR group and OSR group.

Characteristic	TTESR group (*n* = 6)	OSR group (*n* = 18)	*t*/*Z*	*P*
Age, year, mean ± SD	2.2 ± 1.1	2.1 ± 1.0	0.16	0.875
Gender, *n* (%)				1.000*
Male	5 (83.3%)	14 (77.8%)		
Female	1 (16.7%)	4 (22.2%)		
Location site, *n* (%)				1.000*
Left	3 (50%)	9 (50%)		
Right	3 (50%)	9 (50%)		
Length of stay (days)	3.33 ± 1.03	3.2 ± 1.098	−0.326	0.747
Operative time (min)	56.7 ± 20.7	48.1 ± 12.1	−1.258	0.222
Intraoperative blood loss (mL)	2.7 ± 0.8	2.3 ± 0.8	−0.908	0.374
Scoring based on the sum of scar and subjective assessments (9, 10)	6 (5–6)	5 (3–6)	−0.261^&^	0.009
Scoring of overall results (9, 10)	19.2 ± 0.8	18 (17–20)	−1.948^&^	0.051

* Fisher test.

^&^ Mann–Whitney test.

In our series, 24 patients were followed up postoperatively for an average duration of 70.4 ± 24.9 months. During this period, they exhibited a significantly enhanced range of neck motion and rotation. Among them, 6 patients who underwent TTESR presented with aesthetically pleasing skin incisions that healed well and were effectively concealed within the axillary region. With the accumulation of surgical experience, the operative time for TTESR was reduced to a range of 40 to 50 min (Figure 2B).

## Discussion

In conventional OSR, the transverse neck incision has been extensively adopted as the primary approach. However, OSR often leads to visible scarring on the skin overlying lesions in the exposed cervical region, even when these scars are strategically placed within natural skin creases. Such aesthetically unsatisfactory scars may have psychological impacts on patients and cause emotional distress for their families, particularly as children grow older. Consequently, it is imperative to consider cosmetic outcomes during the surgical planning and execution.

With the advancement of minimally invasive techniques and increasing attention to cosmetic outcomes, endoscopically assisted surgery has emerged as a feasible option for pediatric patients. This approach is distinguished by its safety and reduced invasiveness, leading to its growing popularity in pediatric surgical procedures. For instance, minimally invasive endoscopic techniques have been utilized in the management of pediatric neck lesions, such as thyroglossal duct cysts, parathyroid adenomas, dermoid cysts, and ectopic dilated veins (11–13). The endoscopic treatment of CMT was first introduced by Burstein and Cohen in 1998 (14). Complications associated with this technique include injuries to superficial veins and spinal accessory nerve. Over the last few decades, endoscopic operations for CMT have progressed and been documented in previous studies (12, 15–17).

This study introduces a fully transaxillary endoscopic approach that conceals incisions within the axillary fold. This method is characterized by the following features: (1) an airtight operating space is achieved; (2) the axillary incisions are aligned with the natural skin lines, making them virtually imperceptible postoperatively; (3) this procedure employs a two-port technique, which reduces the number of ports from the previous three-port approach. Consequently, this minimizes trauma to the patient and accelerates recovery. Additionally, it eliminates the need for three incisions, thereby reducing spatial competition between the endoscope and surgical instruments. (4) The subcutaneous space is carefully established through blunt dissection beneath the platysma muscle using a clamp inserted through one incision. Guided by endoscope and marked SCM muscle, the clamp initially creates a blunt dissection of the subcutaneous space. Subsequently, a trocar is inserted to progressively expand the subcutaneous space. Compared to traditional OSR, while there were no significant differences in hospital stay duration, operative time, intraoperative blood loss, or total scores in our study, the TTESR still demonstrated superior subjective satisfaction, as evidenced by a significant difference in the sum of scar and subjective assessments. To the best of our knowledge, this is the first study to compare the efficacy of endoscopic techniques with OSR for treating CMT.

One potential limitation of this technique is its limited applicability to infants and young children, as the majority of patients who underwent TTESR in our series were under 4 years of age. Based on our clinical experience, establishing a cavernous working space becomes progressively more challenging with increasing patient age. The surgical correction for patients with CMT is ideally performed before the age of 4, prior to the onset of facial asymmetry and plagiocephaly (18–20). The strong preference for TTESR in this age group can be attributed to its excellent outcomes, minimally invasive nature, and superior cosmetic results. Consequently, we recommend TTESR as the preferred treatment option for patients under the age of 4 when conservative management proves ineffective. Previous ultrasonography studies have demonstrated that fibrosis levels are directly correlated with age, older children typically exhibit higher degrees of fibrosis (2). Therefore, for older children, a retroauricular approach or a combination of the endoscopic transaxillary approach with a micro-neck incision may be considered as an effective alternative (21).

TTESR is associated with several risks, notably the potential for vascular injury. Consequently, meticulous dissection is essential during the procedure. It is advisable to meticulously dissect the SCM muscle fascicle by fascicle and prevent hematoma formation through careful electrocoagulation. Operators must remain vigilant regarding the vascular sheath, as immediate operative intervention may be required in the event of sudden hemorrhage. Frequent manual palpation facilitates accurate navigation of the correct direction and confirmation of complete SCM muscle dissection. Postoperative complications such as subcutaneous emphysema and metabolic disorders resulting from CO_2_ absorption should also be considered. Applying pressure dressing in the axilla post-surgery is recommended. In our series, no cases of infection were observed.

It is imperative to emphasize that proficient endoscopic skills are essential for the successful execution of TTESR. The operator must possess a comprehensive understanding of neck anatomy and demonstrate advanced proficiency in endoscopic techniques. Occasionally, the smoke produced by electrocoagulation during surgery may obscure the operative field, thereby increasing the duration of the procedure. To mitigate this issue, the 3 mm trocar can be maintained in a semi-open state. It should be pointed out that this approach does not compromise the integrity of the subcutaneous space. This procedure should only be carried out when smoke significantly impairs visual acuity. In our initial series of three cases, the operative time was longer but subsequently decreased to a range of 40–50 min. Once the learning curve was completed, the operative time in our series was effectively reduced.

Certain limitations were identified in this study. Firstly, the sample size in our study was relatively limited, which suggests the need for a larger cohort to validate the reliability of the data. Secondly, the majority of patients who underwent TTESR in our series were younger children, reflecting a potential lack of surgical experience with older pediatric patients. Lastly, it is important to highlight that OSR procedures were conducted by different surgical teams, which may have introduced potential statistical bias.

## Conclusions

The findings of our study demonstrate the effective release of SCM muscle tension and the achievement of favorable cosmetic outcomes through TTESR, positioning it as a promising alternative technique for pediatric patients with CMT.

## Data Availability

The raw data supporting the conclusions of this article will be made available by the authors, without undue reservation.
